# Afatinib plus osimertinib in the treatment of osimertinib-resistant non-small cell lung carcinoma: a phase I clinical trial

**DOI:** 10.1186/s12885-022-10467-w

**Published:** 2023-01-03

**Authors:** Satoru Miura, Yasuhiro Koh, Koichi Azuma, Hiroshige Yoshioka, Kenichi Koyama, Shunsuke Teraoka, Hidenobu Ishii, Kayoko Kibata, Yuichi Ozawa, Takaaki Tokito, Jun Oyanagi, Toshio Shimokawa, Takayasu Kurata, Nobuyuki Yamamoto, Hiroshi Tanaka

**Affiliations:** 1 Department of Internal Medicine, Niigata Cancer Center Hospital, 2-15-3 Kawagishicho, Chuo-ku, Niigata, Niigata 951-8566 Japan; 2 Internal Medicine III, Wakayama Medical University, 811-1, Kimiidera, Wakayama, 641-8509 Japan; 3 Center for Biomedical Sciences, Wakayama Medical University, 811-1, Kimiidera, Wakayama, 641-8509 Japan; 4 Division of Respirology, Neurology, and Rheumatology, Department of Internal Medicine, Kurume University School of Medicine 67 Asahi-machi, Kurume, 830-0011 Japan; 5 Department of Thoracic Oncology, Kansai Medical University Hospital, 3-1, Shinmachi 2 Chome, Hirakata, Osaka, 573-1191 Japan; 6 Clinical Study Support Center, Wakayama Medical University, 811-1, Kimiidera, Wakayama, 641-8509 Japan

**Keywords:** Adverse event, Epidermal growth factor receptor, Maximum tolerated dose, Resistance mechanism, Synergistic effect

## Abstract

**Background:**

Conquering acquired resistance to osimertinib remains a major challenge in treating patients with epidermal growth factor receptor (*EGFR*) mutation-positive non-small-cell lung cancer (NSCLC). Thus, we aimed to determine the safety and efficacy of combination treatment with osimertinib and afatinib for patients with acquired resistance to osimertinib.

**Methods:**

This open-label phase I study was a feasibility study of the combination of afatinib and osimertinib for patients with advanced *EGFR*-positive NSCLC who had progressive disease after receiving osimertinib. The primary endpoint was to determine the maximum tolerated dose (MTD). We enrolled patients who received afatinib at three different dose levels (level 1, 20 mg; level 2, 30 mg; level 3, 40 mg) combined with osimertinib at a standard dose of 80 mg once per day.

**Results:**

Thirteen patients were enrolled in this study. The MTD was defined as 30 mg afatinib when combined with daily oral administration of osimertinib (80 mg). The most frequent adverse events were diarrhea (76.9%), anemia (76.9%), and rash (69.2%). Considering the toxicity profiles during all treatment periods, the recommended oral dose of afatinib was determined as 20 mg daily, with an osimertinib dose of 80 mg. For all evaluable patients (*n* = 12), the response rate was 7.7% and the disease-control rate was 46.2%.

**Conclusion:**

Combination therapy with osimertinib and afatinib was tolerable; however, the synergistic effect of afatinib with osimertinib may be limited in osimertinib-resistant patients.

**Trial registration:**

Japan Registry of Clinical Trials ID: jRCTs051180008, registered date: 08/11/2018.

## Introduction

Molecularly targeted therapies have emerged as essential treatment modalities for non-small cell lung cancer (NSCLC). Somatic mutations in the epidermal growth factor receptor (EGFR) tyrosine kinase domain have been reported as definitive biomarkers for predicting the efficacy of *EGFR*-tyrosine kinase inhibitors (EGFR-TKIs) [1]. EGFR-TKI is the standard initial treatment option for patients with active *EGFR* mutations. Notably, in the FLAURA trial, osimertinib demonstrated superior efficacy to gefitinib or erlotinib for the initial treatment of patients with NSCLC [2]. Acquired resistance to initial osimertinib therapy remains the primary challenge for treating patients with *EGFR* mutation-positive NSCLC.

The mechanisms underlying osimertinib resistance can be divided into *EGFR*-dependent and -independent mechanisms [3]. The *EGFR*-independent mechanisms include activating alternative bypass-signaling pathways (such as in MET proto-oncogene receptor tyrosine kinase [*MET*] and *HER2*), oncogene fusions, or histologic transformations. Combination therapy with driver-matched inhibitors (e.g., osimertinib plus *MET* inhibitors for *MET* amplification) has been investigated as an alternative treatment for patients with bypass-signaling resistance mechanisms [4]. The *EGFR*-dependent mechanisms involve various mutations. The secondary *EGFR* C797S mutation has been characterized as the most frequent resistance mutation in *EGFR*-dependent mechanisms, accounting for 10–26% of patients in the second-line population or 7% in the first-line population [5].

Interestingly, preclinical data suggest that treatment with a combination of osimertinib and first/second-generation EGFR-TKIs can overcome the acquired C797S mutation [6]. Based on these preclinical data, concurrent EGFR-TKI therapy has been investigated to overcome this resistance mechanism (NCT03122717, NCT03810807, and NCT03944772). Other co-existing *EGFR* mutations are potentially sensitive to second-generation EGFR-TKIs, especially afatinib [7]. Afatinib, an irreversible EGFR-TKI, shows broad-range antitumor activity against *EGFR* mutations including the uncommon ones [8] [9]. Therefore, we considered that adding afatinib to osimertinib might help overcome *EGFR*-dependent osimertinib resistance, including the secondary C797S mutation and other co-existing *EGFR* mutations. Based on this rationale, we conducted a phase I trial to investigate the safety and efficacy of osimertinib and afatinib combination therapy in patients with acquired resistance to osimertinib.

## Patients and methods

### Study design

This was an open-label, multicenter, dose-finding, phase I study, with a standard 3 plus 3 dose-escalation design. Participating institutions include four university hospitals and Cancer centers in Japan. This study was conducted in accordance with the principles of the Declaration of Helsinki. All patients provided written informed consent before enrolment in the study. The study protocol was approved by the Certified Review Board of Wakayama Medical University. This study was registered on the clinical trials site of the Japan Registry of Clinical Trials (jRCTs051180008, registered date: 08/11/2018).

### Patients

The eligibility criteria were as follows: age ≥ 20 years and histologically or cytologically confirmed advanced NSCLC with EGFR mutations; an Eastern Cooperative Oncology Group performance status (ECOG-PS) of 0 or 1; acquired resistance to osimertinib; patients who could be treated with 80 mg osimertinib without dose reduction; having at least one or more measurable lesion according to Response Evaluation Criteria in Solid Tumors (RECIST) version 1.1; and provision of written informed consent for study participation.

The key exclusion criteria were as follows: unresolved adverse events from previous treatment; a medical history of interstitial lung disease (ILD), drug-induced lung disease, symptomatic brain metastasis, or leptomeningitis; bone metastasis to be treated by surgery or radiation therapy; histologically confirmed small cell carcinoma transformation as a resistance mechanism; uncontrollable pleural, peritoneal, or pericardial effusion; and prior thoracic radiotherapy within the preceding 2 weeks.

### Study objectives and plan

The primary endpoint was the maximum tolerated dose (MTD) based on dose-limiting toxicities (DLTs) recorded over 28 days. DLTs were defined as any of the following adverse events despite adequate management: persistent Grade 4 neutropenia or thrombocytopenia lasting 4 days; Grade ≥ 3 febrile neutropenia; Grade ≥ 3 diarrhea; persistent Grade 2 diarrhea lasting 4 days; Grade ≥ 3 rash and/or paronychia; Grade ≥ 3 mucositis; persistent Grade 2 mucositis lasting 7 days; persistent Grade ≥ 2 nausea lasting 4 days; and Grade ≥ 2 interstitial pneumonitis.

This open-label phase I study was planned as a standard 3 + 3 dose-escalation study. The enrolled patients were treated with afatinib at three different doses in combination with osimertinib at a standard dose of 80 mg once per day. The starting dose of afatinib was 20 mg once per day, with planned dose escalation at the subsequent dose levels of 30 and 40 mg once per day. The MTD was the highest dose level at which < 33% of the patients experienced DLTs during 28 days (the observation period). If one or two out of three patients experienced DLTs, then three more patients were treated with the same dose. If no more than two patients experienced DLTs among six patients, then the dose was escalated to the next level. The secondary endpoints included the overall response rate (ORR), survival outcomes, and biomarker analysis using liquid biopsy.

### Safety and efficacy assessment

Treatment-related toxicities were assessed according to the Common Terminology Criteria for Adverse Events, version 4.0. The MTD was determined based on toxicity during the observation period. After the observation period, the overall toxicity profiles were also considered to determine the MTD and recommended dose (RD). Tumor response and progression-free survival (PFS) were assessed using RECIST version 1.1 as secondary endpoints. Overall survival times were also assessed. The osimertinib-free interval was defined as the period between the termination of prior osimertinib therapy and enrollment in this study.

### Cell-free DNA (cfDNA) analysis using plasma sampling

Peripheral blood samples were collected from patients into K2 EDTA vacutainers (Becton Dickinson, Franklin Lakes, NJ, USA) before administering the combination therapy. Plasma was isolated immediately after drawing blood and stored at − 80 °C until use. Subsequently, cfDNA was extracted from the plasma samples and used for next-generation sequencing (NGS) library construction with the AVENIO ctDNA Surveillance Kit (Roche Diagnostics, Basel, Switzerland) to assess genetic alterations in 197 cancer-related genes. Sequencing was performed using the NextSeq 500 System (Illumina, San Diego, CA, USA), followed by mutation analyses using the Avenio Oncology Analysis Server (Roche Diagnostics).

## Results

### Patient characteristics

From March 2018 to September 2019, 13 patients were enrolled from four participating institutions in Japan. Table 1 shows the background information of the 13 eligible patients. Six patients were enrolled in the level 1 cohort, whereas seven patients were in the level 2 cohort. The mean age of the patients was 67 years. Nine patients (69.2%) were females and six patients (46.2%) were non-smokers. Three patients had recurrent postoperative disease. All patients had an ECOG-PS of 0 or 1 and had adenocarcinoma histology. We detected exon 19 deletions, exon 19 deletions plus T790M, L858R, L858R plus T790M, and an uncommon *EGFR* mutation in four, two, four, two, and one patients, respectively. All of these patients had been treated with 80 mg osimertinib monotherapy without dose reduction. Seven patients (53.8%) were treated with platinum-based chemotherapy before enrollment in this study. Two patients (15.4%) were enrolled in this study after receiving first-line osimertinib therapy. The details of the patients’ treatment histories before enrollment are summarized in Additional file 1.

**Table 1:** Patient characteristics

*n*			(%)
Age, years	Median	67	
	Range	50–76	
Sex, *n* (%)	Male	4	(30.8)
	Female	9	(69.2)
Smoking status	Never	6	(46.2)
	Current/former	7	(53.8)
ECOG-PS, *n* (%)	0	5	(38.5)
	1	8	(61.5)
Histology, *n* (%)	Adenocarcinoma	13	(100)
Clinical stage, *n* (%)	IVA	4	(30.8)
	IVB	6	(46.2)
	Recurrence	3	(23.0)
EGFR mutation, *n* (%)	Exon 19 deletion	4	(30.8)
	+ T790M	2	(15.4)
	Exon 21 L858R	4	(30.8)
	+ T790M	2	(15.4)
	G719C + S768I	1	(7.7)
Treatment lines, *n* (%)	2nd line	2	(15.4)
	3rd line	5	(38.5)
	≥ 4th line	6	(46.2)

### MTD and toxicity analysis

The DLTs during the observation period at each dose are summarized in Table 2. Six patients were enrolled in the dose-level 1 cohort. One patient in this cohort experienced DLT; Grade 3 diarrhea with Grade 2 dehydration was observed in a 77-year-old woman (patient number 2). Seven patients were enrolled in the dose-level 2 cohort. One patient could not be evaluated because of disease progression during the observation period (patient number 10). Two patients experienced DLTs during the observation period in this cohort. Grade 3 diarrhea was observed in a 76-year-old woman (patient number 13). Persistent grade 2 nausea and vomiting lasting 4 days was observed in a 75-year-old woman (patient number 8). Thus, the frequency of DLTs during the observation period was 33.3% in the dose-level 2 cohort. We also evaluated the MTD after the observation periods. Two more patients experienced intolerable toxicity after the observation period. Based on these toxicity profiles, the MTD was defined as 30 mg afatinib, and the RD was defined as 20 mg afatinib with 80 mg osimertinib.

**Table 2:** Dose-escalation level schema and details of dose-limiting toxicities

Treatment	Level 1	Level 2 ^a^	Level 3
Afatinib (once per day; mg)	20	30	40
Osimertinib (once per day; mg)	80	80	80
Number of DLT evaluable patients	*n* = 6	*n* = 6	*n* = 0
DLT, n (%)	1 (16.7)	2 (33.3)	0 (0)
Details of DLTs	77 years, female: Grade 3 diarrhea	75 years, female: persistent Grade 2 nausea/vomiting 76 years, female: Grade 3 diarrhea	–
Additional toxicity profile after the observational periods	–	50 years, female: persistent Grade 2 diarrhea 70 years, female: Grade 3 rash	

^a^ One patient could not be evaluated because of disease progression during the observation period (patient number 10)

### Adverse events

The toxicity profiles for the entire study period are summarized in Table 3. Frequent adverse events (≥25%) included anemia (76.9%), diarrhea (76.9%), skin rash (69.2%), anorexia (38.5%), and oral mucositis (38.5%). The main Grade 3 toxicities were diarrhea (15.4%), hyponatremia (15.4%), anorexia (7.7%), skin rash (7.7%), and fatigue (7.7%). All adverse events were resolved after temporal discontinuation or dose reduction. No cases of ILD or treatment-related death occurred.

**Table 3:** Adverse events during the study period

	All patients (*n* = 13)				Level 2 (*n* = 7)			
	All Gr		Gr 3–4		All Gr		Gr 3–4	
	n	(%)	n	(%)	n	(%)	n	(%)
Neutropenia	1	(7.7)	0	(0)	1	(14.3)	0	(0)
Anemia	10	(76.9)	0	(0)	4	(57.1)	0	(0)
Thrombocytopenia	2	(15.4)	0	(0)	1	(14.3)	0	(0)
Anorexia	5	(38.5)	1	(7.7)	3	(42.9)	0	(0)
Nausea	2	(15.4)	0	(0)	1	(14.3) ^a^	0	(0)
Vomiting	3	(12.1)	0	(0)	2	(28.6) ^a^	0	(0)
Diarrhea	10	(76.9)	2	(15.4)	6	(85.7)	1	(14.3) ^a^
Oral mucositis	5	(38.5)	0	(0)	1	(14.3)	0	(0)
Paronychia	2	(15.4)	0	(0)	1	(14.3)	0	(0)
Skin rash	9	(69.2)	1	(7.7)	4	(57.1)	1	(14.3)
Fatigue	1	(7.7)	1	(7.7)	1	(14.3)	1	(14.3)
Increased ALT	1	(7.7)	0	(0)	1	(14.3)	0	(0)
Increased AST	1	(7.7)	0	(0)	1	(14.3)	0	(0)
Increased creatinine	2	(15.4)	0	(0)	1	(14.3)	0	(0)
Hyponatremia	1	(7.7)	2	(15.4)	1	(14.3)	1	(14.3)

Abbreviations: *Gr* grade, *AST* aspartate aminotransferase, *ALT* alanine aminotransferase

^a^ DLTs in level 2 cohort comprised Grade 2 nausea and vomiting (*n* = 1), and Grade 3 diarrhea (*n* = 1)

### Efficacy

**Fig. 1:**
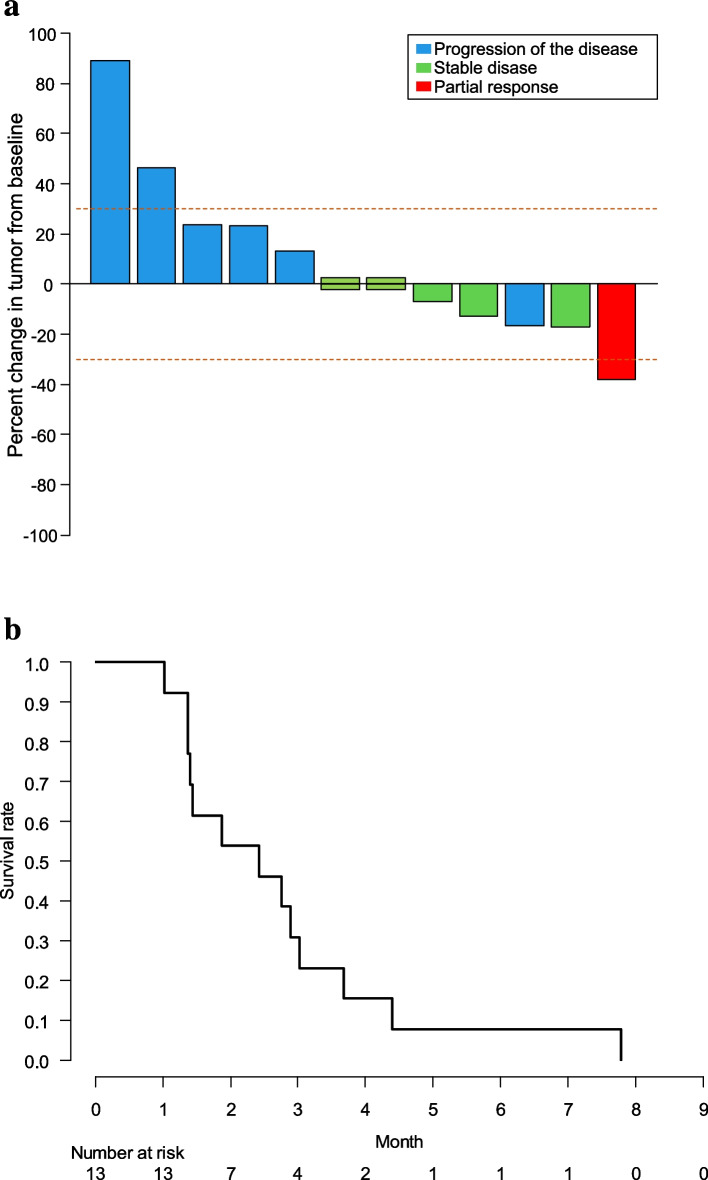
Efficacy analyses of combination treatment with afatinib and osimertinib. (**a**) Waterfall plot of evaluable patients (*n* = 12). The overall response rate was 7.7%. (**b**) Kaplan–Meier curve analysis of PFS. The median PFS duration was 2.4 months

Among the 13 patients enrolled in this study, one patient showed a partial response (PR) and 5 patients showed stable disease (SD) with an ORR of 7.7% (95% confidence interval [CI]: 0.2–36.0%) and a disease-control rate of 46.2% (95% CI: 19.2–74.9%) (Fig. 1a). The patients who achieved a PR were treated with dose level 2. The median PFS was 2.4 months (95% CI: 1.4 to not reached), and 3- and 6-month PFS rates were 30.8 and 7.7%, respectively (Fig. 1b). The median overall survival time was 25.4 months (95% CI: 4.6 to not reached).

### Mutation analysis using liquid biopsy

**Fig. 2:**
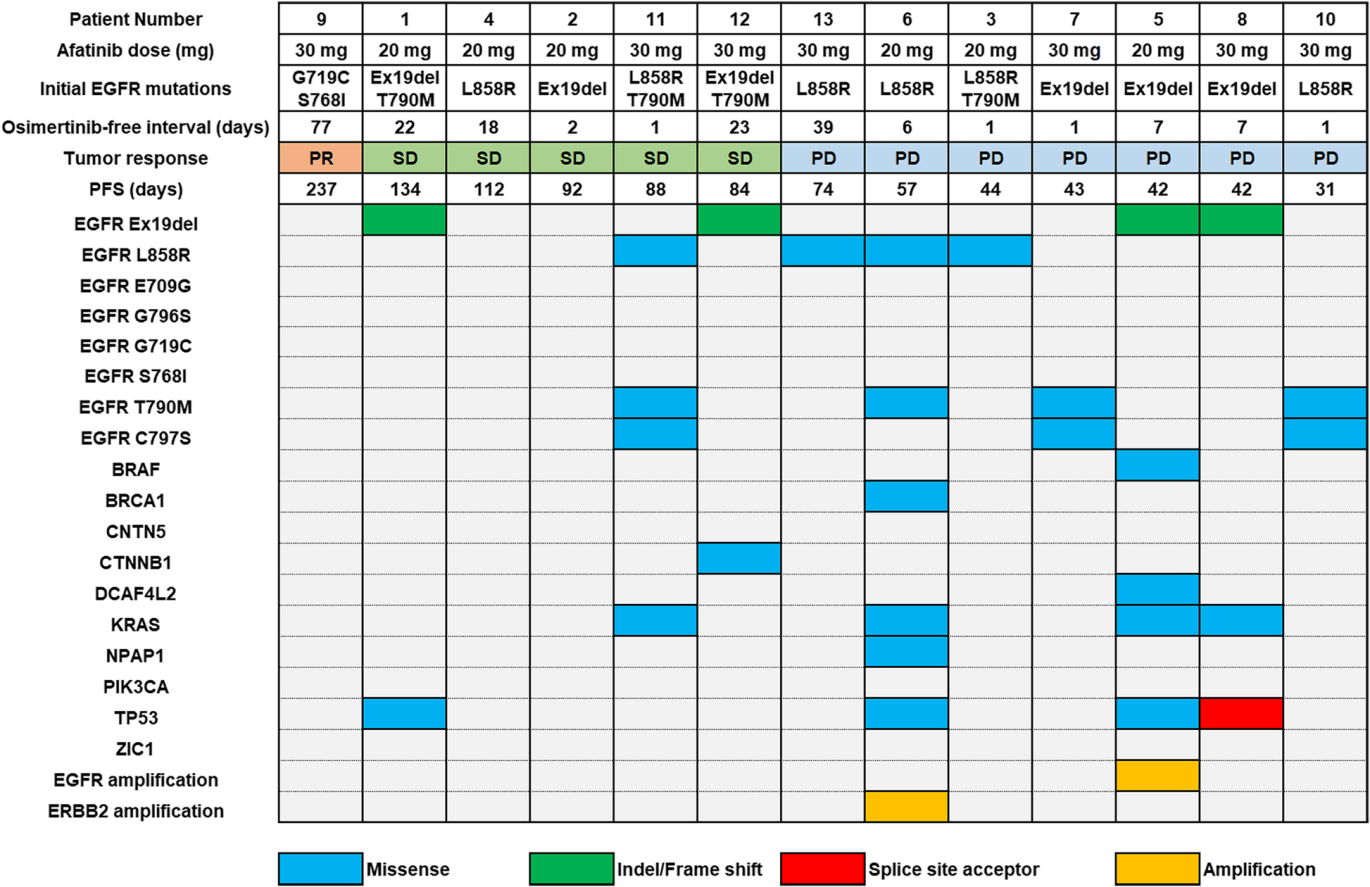
Liquid biopsy results of gene-alteration or -amplification analysis before initiating treatment

Gene alteration and -amplification were analyzed by performing liquid biopsy assays (Fig. 2). *EGFR* mutations were detected in 10/13 (77%) cfDNA samples, including T790M/*cis*-C797S mutations before treatment (patients numbers 7, 10, and 11). Other mutations were detected in six patients before treatment; five of whom had *TP53* and/or *KRAS* mutations, and a variety of compound mutations and/or amplifications were detected in three patients. Amplification of *EGFR* or *ERBB2* was observed in two patients (patients numbers 5 and 6). No relevant associations were observed among the tumor response, PFS, and specific mutation. The patients who partially responded to this combination therapy did not have any gene alterations before treatment using this combination (patient number 9).

## Discussion

This is the first prospective phase I study to investigate the effectiveness of afatinib combined with 80 mg osimertinib. The MTD was defined as 30 mg afatinib combined with 80 mg osimertinib. Based on the toxicity profiles found during all study periods, 66.6% of the patients treated with the MTDs of afatinib and osimertinib experienced DLTs or DLT-equivalent toxicity, such as persistent Grade 2 diarrhea lasting 4 days or Grade 3 rash, respectively. Therefore, the RD for further study was defined as 20 mg afatinib combined with 80 mg osimertinib.

Data regarding the adequate dose of afatinib for concomitant use with 80 mg osimertinib are limited. One case report suggested that 20 mg afatinib with 80 mg osimertinib was well tolerated and that it reduced the frequency of the G724S clone, as assessed by the plasma cell-free DNA testing [10]. In our trial, the MTD and RD of afatinib combined with 80 mg of osimertinib were 30 mg and 20 mg, respectively. The most frequent drug-related toxicities were diarrhea (76.9%), skin rash (69.2%), anorexia (38.5%), and oral mucositis (38.5%). These toxicities were manageable, especially in the 20 mg dose cohort. Low-dose afatinib therapy has already been investigated in several studies. A prospective phase II study using low-dose afatinib monotherapy reported that its clinical efficacy was comparable to that of standard-dose afatinib, without severe toxicities, for EGFR mutation-positive NSCLC [11, 12]. Considering these prior reports, the combination of 20 mg afatinib and 80 mg osimertinib will be a reasonable option for further investigations.

Osimertinib is superior to first-generation EGFR-TKIs used in the first-line treatment of EGFR mutation-positive advanced NSCLC [2]. Despite the high response rate and long PFS, many patients acquire resistance to osimertinib. Understanding the mechanisms of acquired osimertinib resistance is a major area of research. Here, we performed gene-mutation analysis to investigate the target efficacy of combination treatment with afatinib and osimertinib via liquid biopsy analysis. The gene mutations associated with *EGFR* were detected in 77% of patients before treatment. Other genetic alterations, such as *KRAS*, *NPAP1*, and *TP53* mutations, were detected in six patients. Liquid biopsy analysis with NGS may be feasible for detecting druggable mutations and may help in evaluating osimertinib resistance. *MET* amplification, *EGFR* C797S mutations, and histologic transformations have been reported as major resistance mechanisms during initial osimertinib therapy [13]. C797 provides the covalent binding site for osimertinib, and mutations in this site induce resistance by interfering with drug-protein binding [14]. Interestingly, prior-generation EGFR-TKIs, such as gefitinib and afatinib, demonstrated sensitivity to C797S mutations [15]. The results of this study suggest that combination treatment with osimertinib and gefitinib or afatinib is a reasonable strategy against the C797S and T790M variants of EGFR. In this study, mutation profiling was performed with plasma samples using NGS. The three patients harboring the T790M/*cis*-C797S mutation pair did not show treatment responses before initiating combination therapy. C797S serves as the binding site for irreversible EGFR inhibitors, including osimertinib. The C797S mutation is considered a drug-resistance mutation with respect to osimertinib [16]. The location of C797S in *cis* or *trans* may have important biological implications [15]. The location of both T790M and C797S in the same allele (*cis*) led to EGFR-TKI resistance in vitro. The three C797S mutations detected in this study were located in *cis* with respect to the T790M mutation. The efficacy of combination treatment with osimertinib and afatinib against T790M/*trans*-C797S mutations remains unclear.

Secondary EGFR mutations or co-existing uncommon *EGFR* mutations, such as L718Q, G724S, L792H/F/Y, F795C, and G796S/R, have been reported to partially contribute to osimertinib resistance [17]. Several reports have investigated the efficacy of other EGFR-TKIs against these tertiary *EGFR* mutations. Afatinib is a second-generation EGFR-TKI with broad-range sensitivity to various *EGFR* gene mutations [18]. Notably, afatinib’s efficacy against secondary or co-occurring mutations in vitro and in vivo, like the G724S point mutation, has been demonstrated [19]. A large retrospective study demonstrated the moderate sensitivity of osimertinib for various uncommon or compound *EGFR* gene mutations [20]. In this milieu, a combination of osimertinib and afatinib therapy is considered an important strategy for investigating the development of treatments for EGFR mutation-positive NSCLC. However, the molecular profiling conducted in this study did not detect uncommon *EGFR* mutations before treatment. The frequency of resistance mechanisms based on additional uncommon *EGFR* mutations might be relatively rare, requiring more participants to evaluate the efficacy of this combination against osimertinib resistance due to co-existing uncommon *EGFR* mutations.

Co-occurring genomic alterations contribute to the heterogeneity of driver-positive NSCLC and *EGFR*-targeted therapy-resistant cases [21]. Notably, *TP53* mutation is a frequently co-occurring mutation in plasma samples showing a negative prognostic value in EGFR-mutation-carrying NSCLC patients. Other genomic alterations co-occurred in patients with *TP53* mutations in our study and they did not respond to the EGFR-TKI combination therapy. The strategy to overcome heterogeneously mutated cancer has not been established. The subgroup analysis of the RELAY trial suggested that the addition of an anti-VEGFR-2 antibody, ramucirumab, to erlotinib might conquer the *TP53*-positive EGFR-mutated NSCLC [22]. The combination with antiangiogenetic agents may potentially overcome *TP53* co-mutation-induced resistance, and further investigation is needed.

Furthermore, RAS-MAPK pathway activations, including *KRAS* or *BRAF,* can induce osimertinib resistance [3]. Four cases carrying mutations in the RAS-MAPK pathway genes were found in this study, and the efficacy of combination therapy was in 1 case of SD (No. 11) and 3 cases of PD (Nos. 5, 6, and 8). Further investigation is warranted to overcome these complex resistant mechanisms by combining multiple inhibitors, like KRAS allosteric inhibitors, BRAF inhibitors, and MEK inhibitors.

We detected an alternative resistant pathway due to gene amplifications, like in *EGFR* and *ERBB2*. *ERBB2* amplification is an acquired resistance mechanism, with a frequency of 2 and 5% in first- and second-line settings for osimertinib, respectively [3]. Patients with *ERBB2* amplification in our study did not respond to the EGFR-TKI combination therapy including the pan-*HER* inhibitor, afatinib. One reason may be that this patients (No. 6) had additional complex resistance mechanisms, like those conferred by *BRCA-1*, *KRAS*, NPAP-1, and *TP53*. Additionally, in vitro results suggested that the combination of afatinib and cetuximab may be needed to conquer the resistance due to *ERBB2* amplification. Osimertinib resistance is highly diverse, and often associated with multiple gene alterations; overcoming resistance using simple combination therapy is limited. Furthermore, re-biopsy of tumors in this setting is also difficult, and the realization of precise individualized treatment using liquid biopsy is indispensable for overcoming resistance.

A key limitation of this study was the small number of participants. We could not evaluate the efficacy of the combination therapy in patients harboring T790M/*trans*-C797S mutations or co-existing uncommon *EGFR* mutations. In addition, we could not evaluate the osimertinib-resistant status in patients who achieved a PR to osimertinib plus afatinib after acquiring resistance to osimertinib monotherapy because these patients might be “non-shedders” of cfDNA. These findings indicate challenges in conquering heterogeneous EGFR-TKI resistance based on conclusions drawn from studies with a small number of patients. Furthermore, some biases could have affected the treatment outcomes in this setting. For example, in the subgroup analysis of the AURA3 trial, the mutation analysis determined via liquid biopsy showed that detectable plasma T790M status could be a prognostic factor [23]. Furthermore, high tumor burden is a predictor of cfDNA shedding and poor survival [24]. Notably, in our study population, three patients (Nos. 2, 4, and 9), in whom gene mutations or amplifications were not detected, achieved PR or SD with favorable PFS durations. Considering that undetected cfDNA may be a prognostic factor, we should evaluate this result considering selection bias. Second, the TKI-free interval has been reported as a key prognostic factor for PFS in the EGFR-TKI re-administration strategy [25]. Indeed, the osimertinib-free interval was relatively longer (77 days) in a patient showing a PR (patient number 9) than in other patients. It is difficult to avoid these biases in a setting of osimertinib resistance. A reasonable strategy for this EGFR-TKI combination is administration to treatment-naïve patients to delay resistance. The use of gefitinib and osimertinib in treatment-naïve NSCLC patients with *EGFR-*active mutations is being studied (NCT03122717). The results of the present study demonstrated the safety and feasibility of the combination of 20 mg afatinib and 80 mg osimertinib in a late-line setting. Further investigation of this combination in first-line therapy for patients with NSCLC and active *EGFR* mutations is warranted.

## Conclusions

The combination of osimertinib and afatinib was tolerable; however, the additional antitumor effect of afatinib on osimertinib may be limited in patients with osimertinib resistance.

## Data Availability

Raw data for this study were generated at Wakayama Medical University. The datasets generated and/or analyzed during the current study are not publicly available but are available from the corresponding author upon reasonable request.

## Abbreviations

ALT: Alanine aminotransferase
AST: Aspartate aminotransferase
cfDNA: Cell-free DNA
CI: Confidence interval
DLT: Dose-limiting toxicity
ECOG: Eastern Cooperative Oncology Group
EGFR: Epidermal growth factor receptor
Gr: Grade
ILD: Interstitial lung disease
MET: MET proto-oncogene receptor tyrosine kinase
MTD: Maximum tolerated dose
NGS: Next-generation sequencing
NSCLC: Non-small cell lung cancer
ORR: Overall response rate
PFS: Progression-free survival
PR: Partial response
PS: Performance status
RD: Recommended dose
RECIST: Response Evaluation Criteria in Solid Tumors
SD: Stable disease
